# Improved Faster R-CNN for the Detection Method of Industrial Control Logic Graph Recognition

**DOI:** 10.3389/fbioe.2022.944944

**Published:** 2022-08-04

**Authors:** Shilin Wu, Yan Wang, Huayu Yang, Pingfeng Wang

**Affiliations:** ^1^ Hubei Key Laboratory of Digital Textile Equipment, Wuhan Textile University, Wuhan, China; ^2^ School of Mechanical Engineering and Automation, Wuhan Textile University, Wuhan, China

**Keywords:** SAMA logic diagram, faster R-CNN, K-means, non-maximal value suppression algorithm, improved faster R-CNN

## Abstract

In the process of developing the industrial control SAMA logic diagram commonly used in the industrial process control system, there are some problems, that is, the size of logic diagram elements is small, the shape is various, similar element recognition is easily confused, and the detection accuracy is low. In this study, the faster R-CNN network has been improved. The original VGG16 network has been replaced by the ResNet101 network, and the residual value module was introduced to ensure the detailed features of the deep network. Then the industrial control logic diagram dataset was analyzed to improve the anchor size ratio through the K-means clustering algorithm. The candidate box screening problem was optimized by improving the non-maximum suppression algorithm. The elements were distinguished *via* the combination of the candidate box location and the inherent text, which improved the recognition accuracy of similar elements. An experimental platform was built using the TensorFlow framework based on the Windows system, and the improved method was compared with the original one by the control variable. The results showed that the performance of similar element recognition has been greatly enhanced through an improved faster R-CNN network.

## Introduction

**FIGURE 1:**
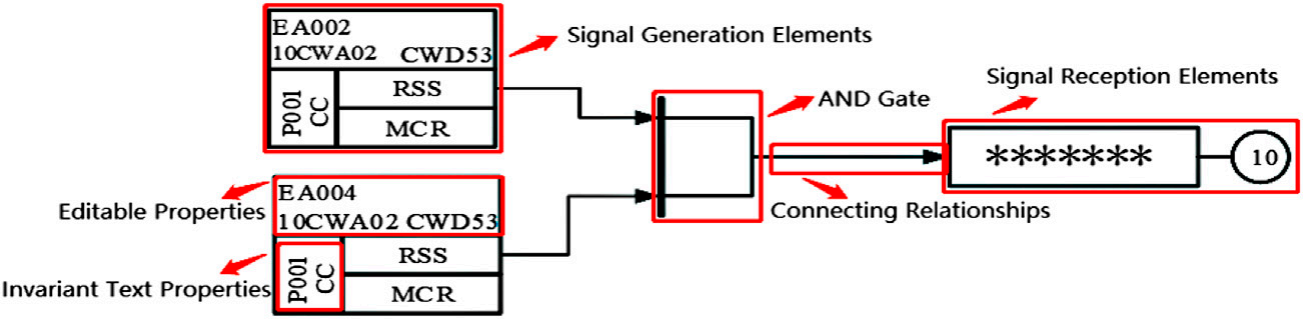
The part of the industrial control logic diagram information.

The industrial control logic SAMA diagram is often used to draw the industrial process control system, which can visually reflect all the control functions and signal processing functions. This kind of industrial control logic diagram is the control system architecture diagram covering most of the instrumentation components in the control system, which is one of the most used process control system drawing examples nowadays. In view of its significance in the manufacturing process, the SAMA diagram needs to be logically verified. In order to solve the problem of low efficiency and time consumption of the original manual verification of the industrial control logic diagram, it is necessary to identify the logic diagram PDF files drawn by designers and transform them into XML file format conforming to the simulation platform specifications. SAMA diagrams include element categories, properties, connection relationships, and sheet properties. The identification of logical elements of SAMA diagrams is the key to the transformation recognition process because both text properties and segment connections are attached to the logical element. Understanding the batch transformation of the logic diagram will greatly improve the verification efficiency. Figure 1 shows the part of the industrial control logic diagram information.

In recent years, engineering drawing identification and verification have also gradually developed in the direction of intelligence. Automatic drawing verification can improve the verification efficiency and save time. The Viola–Jones image classifier structure was used to identify seal images in document images (Matalov et al., 2021). Rho et al. (2020) identified the engineering drawings and extracted the row text in the document to automatically generate the building’s 3D model. Jia (2012) used template matching to identify the tables in the drawing to simplify the identification steps.

When the engineering drawing page is large, it is also very important to use the page segmentation technology to process the drawings in advance. An adaptive over-splitting and merging algorithm to handle document over-segmentation and under-segmentation errors was proposed by Ha et al. (2016). A top-down document segmentation method for identifying paper documents was proposed (Naik et al., 2017). Tong et al. (2013) used the normalized SAD registration method to register the segmented document images. It is also crucial for text recognition in general engineering drawings. Nuzaili et al. (2016) have proposed an enhanced structure perception feature extraction model (PFM) for identifying Arabic characters based on character shape features and point features. Jorge (2012) identified and matched text in paper document images to retrieve the document in the document library. Jose and John (2015) identified Malayalam Braille documents and converted them all into audio output. Li and Stevenson (2017) employed a modified Canny operator to detect edges from images, and then extracted line segments from these edges. Using deep learning technology to make document identification analysis has also become a popular topic in recent years. Liu and Fink Gernot (2018) conducted a special study on document analysis and identification, and found that different neural network algorithms have been used to identify documents.

The traditional detection algorithm adopts manual feature extraction for training, but the extraction process always loses part of the image feature information, resulting in an unsatisfactory recognition effect. Deep learning–based detection technology is more efficient and flexible in facing complex data, making up for the problem of losing image feature information. Due to the advantages of autonomous learning of neural networks, the input original data can extract deeper features. A rapid convolutional neural network for license plate recognition in real-life scenarios was proposed (Liu and Liu, 2014). Navaneeth et al. (2017) proposed a soft-NMS algorithm which could be easily integrated into any object detection pipeline for the non-maximum suppression part of object detection. Sethuraman et al. (2022) used a convolutional neural network to identify images taken by drones and detect field fires. Inkeaw et al. (2022) used a 3D convolutional neural network for brain CT image recognition to determine whether intracranial hemorrhage occurred. Chen et al. (2022) used convolutional neural network analysis to judge the sediment content in rivers and lakes.

With the continuous development of computer technology, deep learning technology is also constantly optimized. Girshick (2014) proposed the R-CNN network, which combined deep learning and candidate region generation. The image feature information candidate box was generated by using a selective research method, then the image features were obtained using a convolutional neural network model, and finally, the linear support vector machine classifier was used for recognition and detection. The Mask R-CNN was modified to identify agricultural pests by Rong et al. (2022). The R-CNN was improved for vehicle traffic sign detection in real-life scenarios by Liang et al. (2022). He et al. (2015) proposed SPP-Net, which could extract feature vectors of the same length by using spatial pyramid pooling layers of different sizes, and it performed well for input images of different sizes. Yi et al. (2019) used SPP-Net to identify peripheral CT images of the spine to judge for medical diagnosis.

Subsequently, Girshick (2015) proposed the fast R-CNN algorithm, which combined the advantages of R-CNN and SPP-Net to train the target classifier and candidate box regression simultaneously. Fast R-CNN improved the identification efficiency of the network model to some extent, but the computational cost was large. The fast R-CNN was used for vehicle category detection in traffic environments (Arora et al., 2022).

This defect was overcome using the faster R-CNN algorithm proposed by Ren et al. (2015). The algorithm innovatively adopted the regional generation network and used the anchor point to generate the candidate box for the feature images extracted by the convolutional layer. Then, the object or the image background in the candidate box was preliminarily determined according to the border regression method in the region generation network. Finally, the processing continues through other network structures. Yang et al. (2022) used faster R-CNN to detect the melting of iron ore powder online, to establish the correspondence between temperature and the speed of melting. Intelligent prediction of the wear position and mechanism of the machine after the modification of faster R-CNN (Wang et al., 2022). Xie et al. (2022) used the residual network to optimize the feature extraction network of faster R-CNN, and the improved network greatly enhanced the accuracy of fault detection. Zhao et al. (2022) used ResNet50 to replace the feature extraction network VGG16 in faster R-CNN to identify cylinder yarn types, which solved the problems of high labor intensity and low efficiency of manual identification of cylinder yarn types.

The aforementioned deep learning methods all used input images to generate regions and then conduct classification regression, which belonged to the two-stage target detection algorithm and lacked real features. Joseph et al. (2015) proposed the YOLO algorithm to strengthen the real-time feature detection of the network. The network grated the input images and made predictions for different grid regions at the same time, directly outputting the regression box and confidence of the predicted regions.

The YOLO algorithm has also been improved to meet the needs of actual target detection scenarios. Jiang et al. (2022) summarized the target recognition and feature selection methods of the YOLO algorithm by comparing the different versions of the information, and proved the sustainability of the improvement of the yolo algorithm. A YOLO v5-based ship target detection algorithm was proposed for ship target detection based on remote sensing images with complex backgrounds by Sun et al. (2022). Mao (2022) improved the YOLO algorithm for real-time pedestrian detection in low light and crowded scenarios.

The two-stage detection algorithm is more accurate than the single-stage detection algorithm. Therefore, this study introduced the faster R-CNN network for the logical graph element recognition. According to the characteristics of the small size of logical elements, different shape proportions, many similar elements, and dense distribution, the network was improved to make it more suitable for the target identification of logical elements in the SAMA logical graph. The improved aspects of the original faster R-CNN network are as follows:

(1) In order to avoid the serious loss of feature information caused by the excessive pooling layer, the original VGG16 network was replaced with the ResNet101 network, and the residual value module was introduced to enrich the deep network features.
(2) The K-means (Peng et al., 2021) clustering algorithm was used to analyze the element dimension scale of the logical graph dataset, and the size scale of the original network anchors was also improved.
(3) The non-maximum suppression algorithm was improved and a linear penalty mechanism was introduced to avoid the detection box being deleted directly due to the high score of the adjacent detection box.
(4) The associated logic element text was introduced to improve the recognition rate of similar logic elements.

## The Creation of Logical Element Dataset

The industrial control SAMA logic diagrams are drawn in strict accordance with industrial standards using professional drawing templates. However, the drawing templates contain more interference information, such as module titles, edge grids, and irrelevant text, which will interfere with the recognition of industrial control logic diagram elements. Therefore, the masking process was adopted to remove the edge grid and irrelevant information from the drawing. In order to ensure the generalization ability of the training model, the method of rotating the image by 180° to enhance the dataset was used.

All the different kinds of logical elements in the dataset under the background of the common logical drawing template are selected for model training. A total of 534 logic diagram images were selected, and the image size was fixed to 1680*1280. A total of 1,068 industrial control logic diagram images were obtained through data enhancement. There were 54 kinds of industrial control logic diagram elements, and 3,564 industrial control logic diagram elements of various categories. The LabelImg tool was used to label the logic elements to get the corresponding XML file.

## The Algorithm Structure of Logical Element Recognition

**FIGURE 2:**
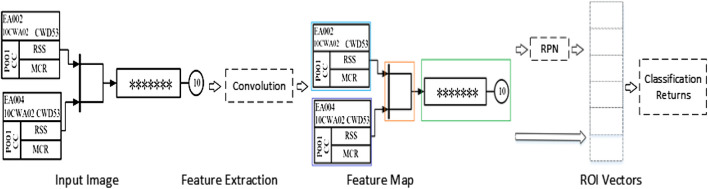
Faster R-CNN basic network framework.

The faster R-CNN network model was selected for the target detection of logical graph elements, and the framework of this network is shown in Figure 2.

The network model used a VGG16 convolutional neural network to process the input image and extract the image area features. Then, the processed images were input into the region proposal network (RPN) which extracted the feature images generated by the network according to the features. Relying on the custom sliding window on the feature graph, nine class target boxes for each position were regarded as the initial detection box. Then, whether the target object or image background was within the anchors was determined, and the anchors that belonged to the target object were coordinated. Finally, the anchor frames generated in the RPN were modified by the target classification and position regression modules. The NMS algorithm was used to handle the problem of selecting the same target for different anchors, and the different anchors needed to be classified to determine the type of target.

## The Optimization of Faster R-CNN

### Improved Feature Extraction Network

The VGG16 network, as a feature extraction network of the faster R-CNN model, has a relatively good performance for detection of targets with clear pixels and large sizes; however, it is not ideal for detecting and identifying logical elements with small image pixel sizes. It was pooled once after each convolution, while the original size of the input image was compressed after pooling, indicating that the pixels of the feature map were gradually decreasing, which will cause the feature information of the logical elements in the deep feature map to be significantly reduced. Therefore, the VGG16 poorly detects logical elements with a small pixel size.

**FIGURE 3:**
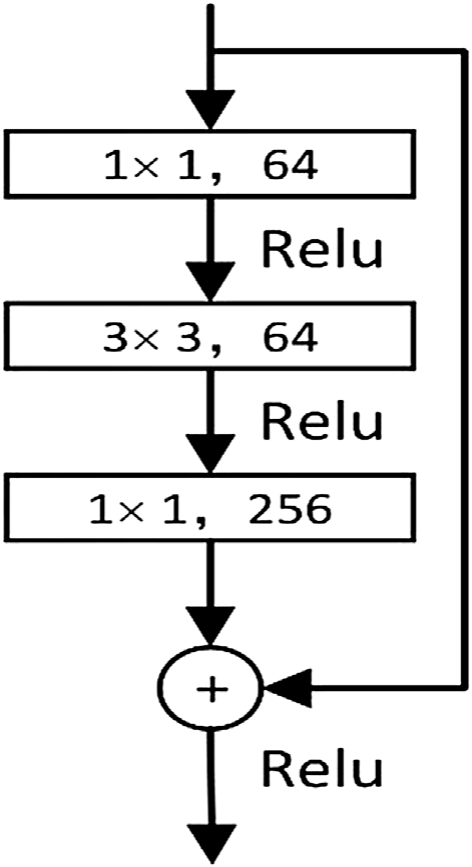
Residual network structure.

In order to solve the problem of decreasing the features of the elements with the number of layers of the network deepening and avoid the gradient disappearance and explosion, the ResNet101 network was used to replace the VGG16 network, and it adopted the residual value blocks and “jump connections.” One “jump connection” was added after every n layer on top of the normal network, thus forming multiple blocks of residual values. The output feature information of the former block and the latter block were combined to ensure the richness of the deep network features. This structure established the mapping relationship by associating the input features and output features between the blocks, ensuring that the image features do not gradually decrease as the network deepens, and the stability of the network performance. The residual block structure of ResNet101 was shown in Figure 3.

### The Optimization of Anchors by K-Means

**FIGURE 4:**
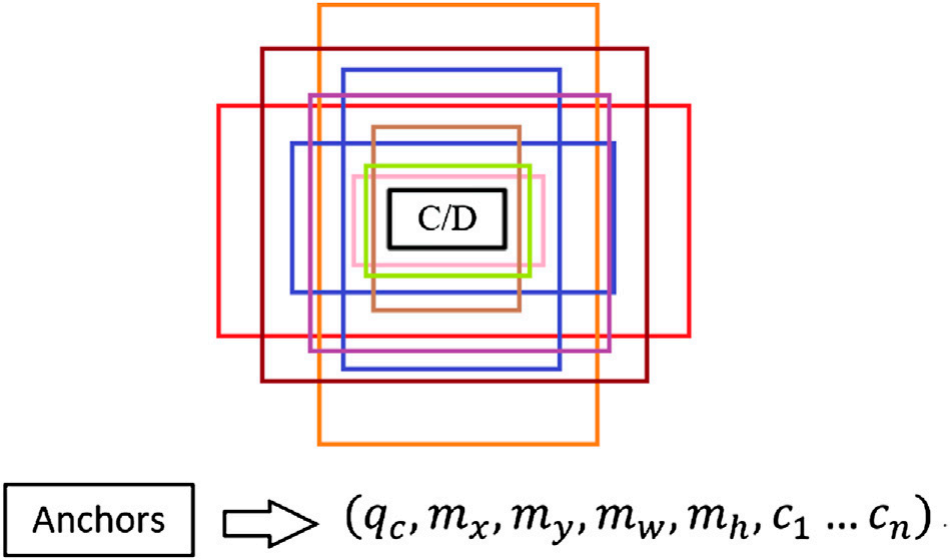
Sample of anchors and area vector.

Anchors are candidate frames defined in the RPN network according to the established dimensions (width and size). Based on the feature information extracted by the convolutional layer and the custom sliding window on the feature map, nine classes of target boxes (anchors) were generated for each position as initial detection boxes. Figure 4 shows an example of the simplified image rasterization with the corresponding nine anchor box cases in the region generation network.

**FIGURE 5:**
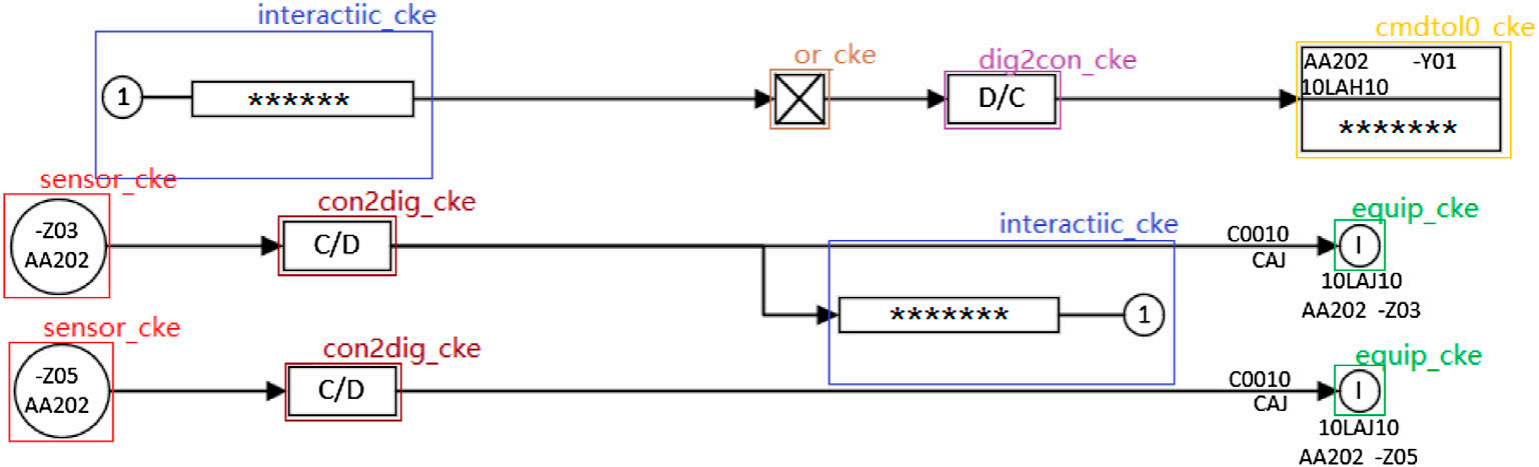
Example of logical diagram detection.

Anchors in traditional RPNs correspond to three scales (128, 256, and 512) and three aspect ratios (1:1, 1:2, and 2:1). These scale sizes were in line with common sense for general object detection, but for logic diagram elements with larger aspect ratios, the ratios did not match the original anchors’ corresponding sizes. These three sizes (1:1, 1:2, and 2:1) in the network training would cause the candidate frame size not to be adaptively and accurately matched with the graphic elements in the industrial control logic SAMA graph, which affected the accuracy of network detection. Figure 5 shows the matching example of the original logic graph detection. The long element type could not make the recognition box fit the logic graph element accurately because the proportion does not match the size.

**FIGURE 6:**
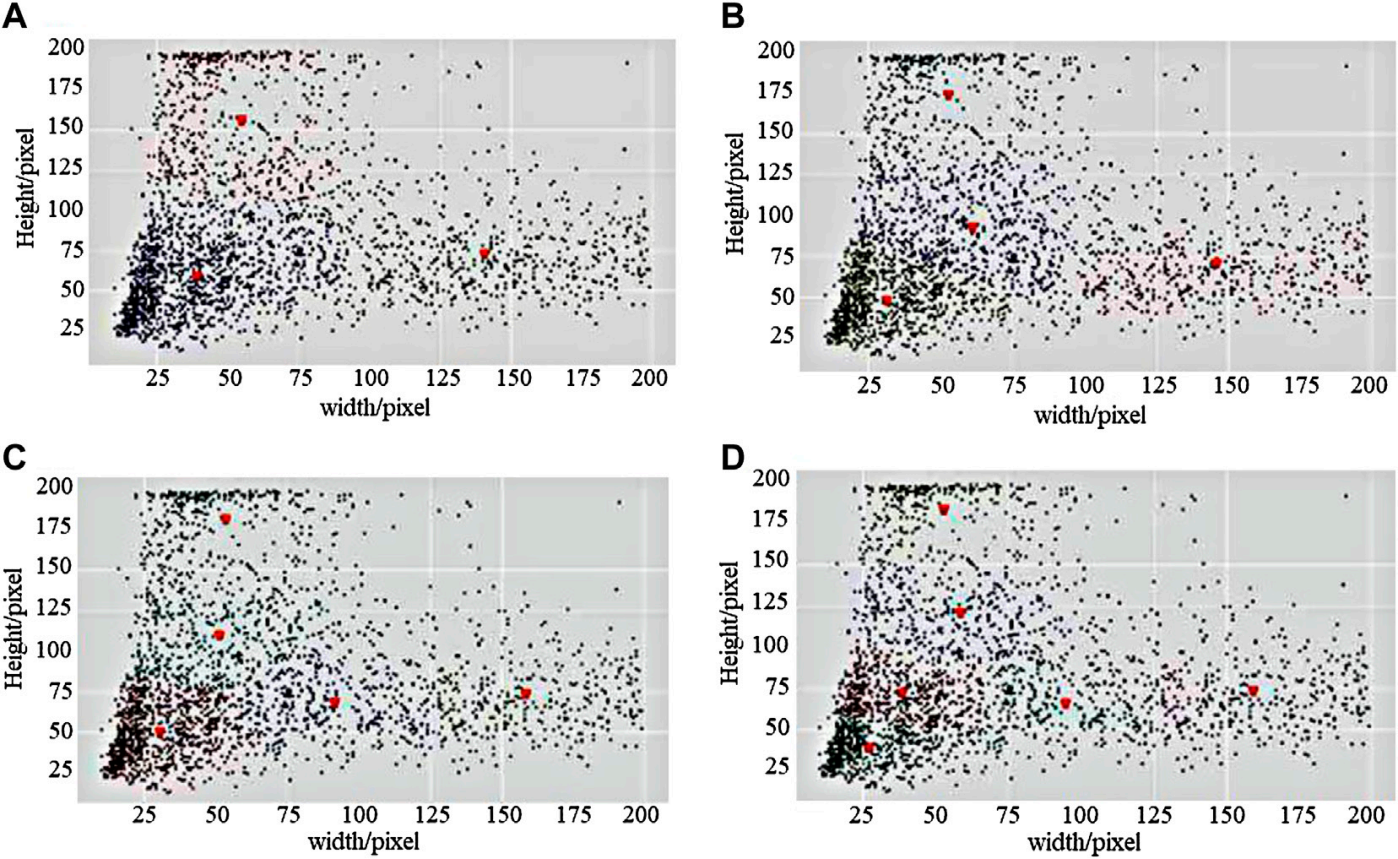
The clustering effect of logical elements dataset. **(A)** K = 3, **(B)** K = 4, **(C)** K = 5, and **(D)** K = 6.

Therefore, the K-means clustering algorithm was adopted for the analysis of the industrial control logic diagram element dataset and the design of anchors that were the most suitable for industrial control logic diagram element size and proportion. The corresponding coordinates of all category logical elements in the XML file were extracted and the sizes of the foreground target were calculated. The length and width of all elements were made into vectors as input to the K-means model. The number of clustering centers was set to 3, 4, 5, and 6 classes, and a comparative analysis of the cluster effect map was performed, according to the actual situation of the dataset. Figure 6 shows the clustering effect of the logical element dataset and the size of most elements was below 100 pixels.

It could be intuitively seen that K = 5 was more suitable for the distribution of sample length and width in the logic map dataset compared with the other three cases of K = 3, K = 4, and K = 6. Therefore, the five cluster center values of K = 5 were set as the five sizes of candidate boxes in the region generation network. The two candidate box ratios of (1:3) and (3:1) were also added according to the sample points around the five cluster centers. The final coordinates of the five cluster centers obtained were (29.358, 47.125), (90.186, 65.324), (50.183, 108.392), (157.524, 71.796), and (51.234, 179.258). According to the clustering results, this experiment used (30, 70, 90, 110, 160) and (1:1, 1:2, 2:1, 1:3, 3:1) as the sizes and ratios, respectively, for improving the existing anchor frames of the RPN network. Table 1 shows the size of anchors before and after improvement.

**TABLE 1:** Size of anchors before and after improvement.

Network	The scale scaling strategies of anchors
Before	(128, 256, 512) + (1:1, 1:2, 2:1)
After	(30, 70, 90, 110, 160) + (1:1, 1:2, 2:1, 1:3, 3:1)

### The Improvement of the NMS Algorithm for the Logical Element Anchors Misdetection

When the feature image was input to the region generation network, this network generated nine classes of target boxes as initial detection boxes for each position based on the custom sliding window on the feature map. Different initial detection boxes had corresponding scores so that there would be multiple candidate boxes for the same detection object location in the same region.

The purpose of using the non-maximum suppression algorithm was to remove the redundant prediction frames, which avoided the multiple calculations of the loss function values. The classical non-maximum suppression algorithm implements the process as follows: the confidence of all ROIs was calculated and arranged in descending order in the process of edge regression. The ROI with the largest confidence was taken. The intersection over union (IOU) of this ROI and all other ROIs was calculated, and if it was larger than the established threshold, the edge was discarded. The aforementioned steps were cycled until all ROIs were finished. The calculation method of IOU was shown in Eq. 1. The flow of the NMS algorithm was shown in Algorithm1.

$$r_{IOU}=\frac{ROI_{\max}\cap ROI_{neighbor}}{ROI_{\max}\cup ROI_{neighbor}}.$$ (1)

Algorithm 1 NMS Algorithm

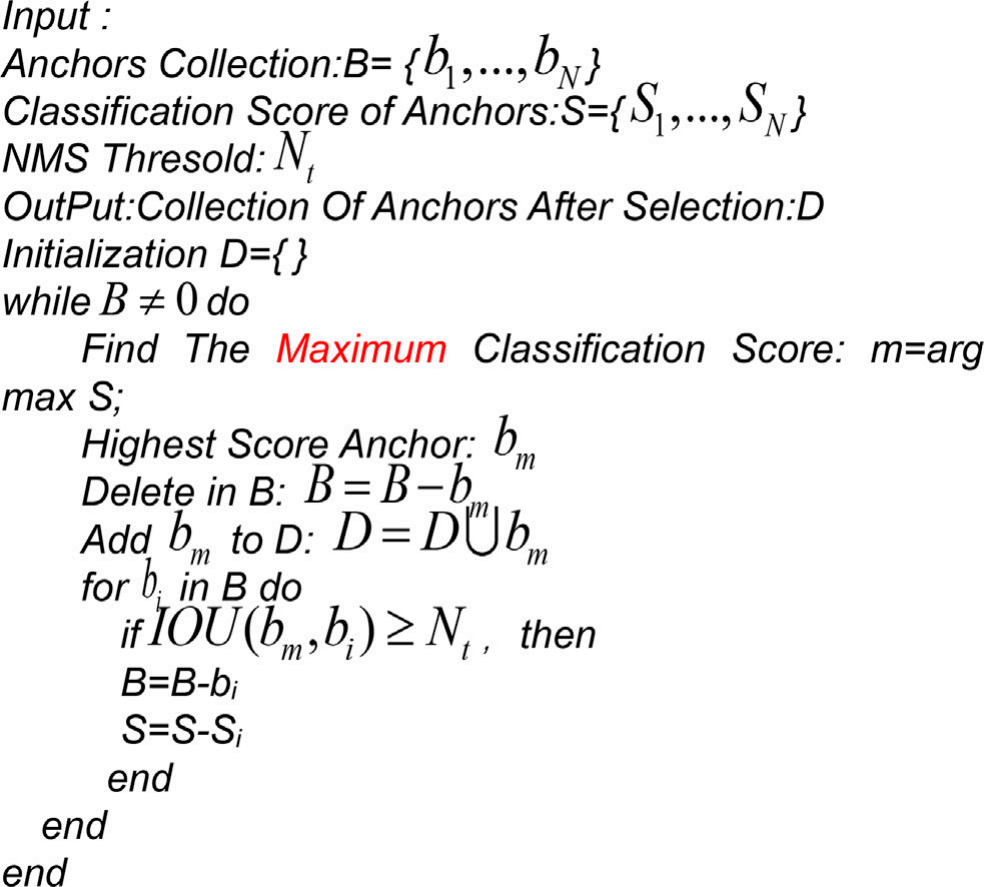

The principle shows that the algorithm has a poor resilience and the following two drawbacks:

(1) The non-maximum suppression algorithm usually retains the anchor box with the highest score. However, there is a situation where the anchor box with a lower score fits the recognized graph element better than the anchor box with a higher score in the process of actual graph element recognition sometimes.
(2) When two logical elements are close to each other, there will be an overlapping of the two candidate frames. If the overlap of the highest scoring frame is greater than the threshold set by the non-maximum suppression algorithm, the candidate frame of one of the elements will be deleted. Especially when the recognition target is dense or there is an overlap, it is easy to cause the phenomenon of missed detection.

To address the aforementioned problems, the starting point of this study is to improve the original non-maximum suppression algorithm by combining it with the Soft-NMS algorithm (Bodla et al., 2017), rather than directly deleting the frames with intersection and ratio scores greater than the set threshold.

A linear coefficient K was introduced based on the combination of the traditional non-maximum suppression algorithm and Soft-NMS. When the intersection over the union of the highest scoring anchor frame and the adjacent anchor frames exceeded the threshold, the scores of the adjacent anchor frames would linearly decrease. The anchor frames that did not exceed the threshold would not be affected. The improved NMS algorithm was shown in Eq. 2.

$$s_{i}=\{\begin{array}{c} s_{i},r_{IOU}\left(M,x_{i}\right)<Value\\ Ks_{i}\left(1-r_{IOU}\left(M,x_{i}\right)\right)r_{IOU}\left(M,x_{i}\right)\geq Value \end{array},$$ (2)

where s_i_ represents the confidence score of the current ROI, *M* is the detection frame with the highest score, *K (*0 < *K* < 1*)* is the elasticity coefficient, x_i_ is the current ROI, and ${\text{r}}_{\text{IOU}}\left(\text{M},{\text{x}}_{\text{i}}\right)$ indicates the intersection ratio of M and ${\text{x}}_{\text{i}}$ .

### The Associated Element Inherent Text Properties for Solving Similar Shape Logical Elements Misdetection

**FIGURE 7:**
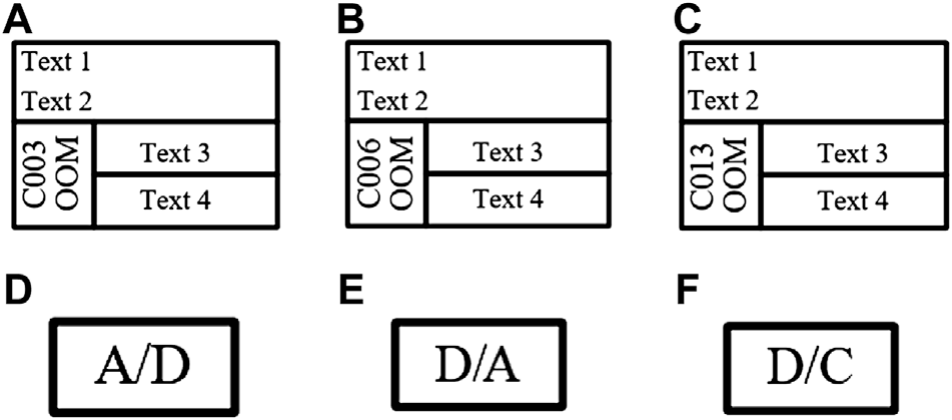
Six common diagram elements in industrial control logic SAMA diagram. **(A)** C003oom, **(B)** C006oom, **(C)** C013oom, **(D)** Ana2Dig, **(E)** Dig2Ana, and **(F)** Dig2Con.

Figure 7 shows six common diagram elements in the industrial control logic SAMA diagram. The logic elements in the industrial control logic diagram library had the same appearance and they could only be distinguished by the different intrinsic text properties contained in the element, which caused some interference to the identification of industrial control logic diagram elements. Therefore, the identification of industrial control logic elements with consistent appearance was wrong.

Therefore, in this experiment, the specific text property features were added to logic diagram elements for distinction between similar elements. After the input logic graph image was pre-processed, the faster R-CNN network completed recognition. If the unique text of each element and the candidate box were matched correctly, then the candidate box was kept. Otherwise, the output candidate box was discarded.

## Experimental Comparison of Improved and Original Faster R-CNN for Logical Element Recognition

This section explores the effects of different experimental strategies on the results to better demonstrate the accuracy of the method on the assay results.

### The Experimental Environment and Evaluation Indicators for Logical Element Recognition

A GTX 1050 Ti GPU supporting parallel computing was used in the experiments. The network training parameters are shown in Table 2. The mAP (mean of average precision) and FPS (frames per second) were used as the evaluation metrics of the network model.

**TABLE 2:** Faster R-CNN training parameters.

Parameter	Setting	Meaning
Learning_rate	0.001	Initial learning rate
Batch_size	48	Number of samples
Max_batches	1000	Maximum iterations number
Weight_decay	0.005	Weight decay value

### Comparative Analysis of Feature Extraction Network for Logical Element Detection Results

The effect of different networks on the accuracy of logical graph element recognition was explored. The control variable method was used to keep the dataset, training steps, and parameter settings, and the experiment results of ResNet101 and VGG16 were compared and analyzed. The initialization settings were performed based on the network parameters given in Table 2. Six commonly used graph elements in Figure 7 were selected for experimental validation, and the experiment results under 1,000 iterations are shown in Table 3. The mAP of the ResNet101 network was improved by 2.6% compared with the VGG16 network, which proved that the ResNet101 network was superior in the detection of industrial control logic graph elements.

**TABLE 3:** Comparison of logical element’s detection results of different feature extraction networks.

Network	Precision/%						mAP (%)
	C003oom	C006oom	C0013oom	Ana2dig	Dig2ana	Dig2con	
ResNet101	92.3	92.7	92.5	87.9	88.3	88.7	92.3
VGG16	91.2	91.1	90.9	85.1	86.2	85.3	89.7

### Anchor Size Validation for Logical Element Recognition

The data clustering analysis was performed on the dataset of industrial control logic graph elements by using K-means clustering to obtain the exact size and scale of the elements. The effectiveness of the different sizes and proportions for improving anchors based on the K-means clustering algorithm was verified. Three sizes (30, 70, 90), (70, 90, 110), and (30, 70, 90, 110, 160) were compared with the original size (128, 256, 512). The sizes and proportions of anchors were validated as shown in Table 4.

**TABLE 4:** Anchors size validation for logical element recognition.

Scale-scaling strategy of the anchors	mAP (%)
(128,256,512) + (1:1,1:2,2:1)	89.7
(128,256,512) + (1:1,1:2,2:1,1:3,3:1)	90.9
(30,70,90) + (1:1,1:2,2:1)	91.4
(30, 70, 90) + (1:1, 1:2, 2:1, 1:3, 3:1)	93.2
(70, 90, 110) + (1:1, 1:2, 2:1)	91.6
(70, 90, 110) + (1:1, 1:2, 2:1, 1:3, 3:1)	92.9
(30, 70, 90, 110, 160) + (1:1, 1:2, 2:1, 1:3, 3:1)	94.6

The highest mAP value (94.6%) was obtained when the combination of (30, 70, 90, 110, 160) + (1:1, 1:2, 2:1, 1:3, 3:1) was chosen in anchors. But more candidate frame size ratios would result in more generated candidate frames and more time-consuming training. The detection time and computational cost should be considered while satisfying the detection accuracy of industrial control logic diagram elements. Therefore, the combination of anchor size ratio (30, 70, 90) + (1:1, 1:2, 2:1, 1:3, 3:1) is more suitable.

### The Validation of the NMS Algorithm on the Detection Results of Logical Elements

In order to verify the effectiveness of the improved NMS algorithm for network detection, the traditional NMS algorithm was compared with the NMS algorithm with the addition of the linear penalty mechanism. The control variable method was used to change only the value of the elasticity coefficient *K* to observe the average accuracy of the algorithm model, and the optimization of the NMS algorithm is shown in Table 5.

**TABLE 5:** NMS algorithm optimization validation for logical element recognition.

Algorithm model		mAP (%)
Original NMS		93.2
Improved NMS	K = 1.0	96.3
	K = 0.95	95.6
	K = 0.8	94.2
	K = 0.6	93.4
	K = 0.3	91.8

When the elasticity factor *K* = 1.0 in the improved NMS algorithm, the network has the highest mAP value (96.3%) which was improved by 3.1% compared with the traditional NMS. The non-maximum suppression algorithm with the addition of a linear penalty mechanism helped counter the problem of missed detection and recognition duplicates in the process of graph element detection.

### Associated Text Inherent Property Validation for Logical Element Recognition

We verified the effectiveness of associated text properties in helping distinguish between appearance-similar logical elements in network detection. The influence of an associated text attribute on the accuracy of logical element recognition was explored. Table 6 shows the experiment results under 1,000 iterations. When elements are associated with text properties, the recognition accuracy of similar elements is higher, such as the precision of C003oom which had improved by 0.4% more the original one, indicating that it is effective in adding the unique text property features of industrial control logic elements as a scheme to distinguish between similar elements.

**TABLE 6:** Associated text intrinsic property validation for logical element recognition.

Associated text	Precision (%)					
	C003oom	C006oom	C0013oom	Ana2dig	Dig2ana	Dig2con
Yes	94.2	93.5	93.1	98.7	97.2	97.4
No	93.8	92.9	92.3	97.2	96.4	95.7

The improved faster R-CNN algorithm was compared with other algorithm models, and each algorithm model training used the existing industrial control logic graph dataset. The experimental results are shown in Table 7.

**TABLE 7:** Comparative analysis of improved faster R-CNN on the detection result of logical elements.

Algorithm model	mAP (%)	FPS
Improved Faster R-CNN	96.3	40.25
Faster R-CNN	89.7	36.32
YOLO_V3	83.4	60.15

The improved faster R-CNN network model had a significant improvement in detection accuracy compared with the original faster R-CNN network and YOLO_V3 network. But the detection speed was still inferior compared with YOLO_V3, which needs to be improved. Compared with the traditional faster R-CNN, the improved algorithm improved the mAP value by 6.6% and the FPS reached every 40.25 frames, which indicated the improved Faster R-CNN network model had better performance.

**FIGURE 8:**
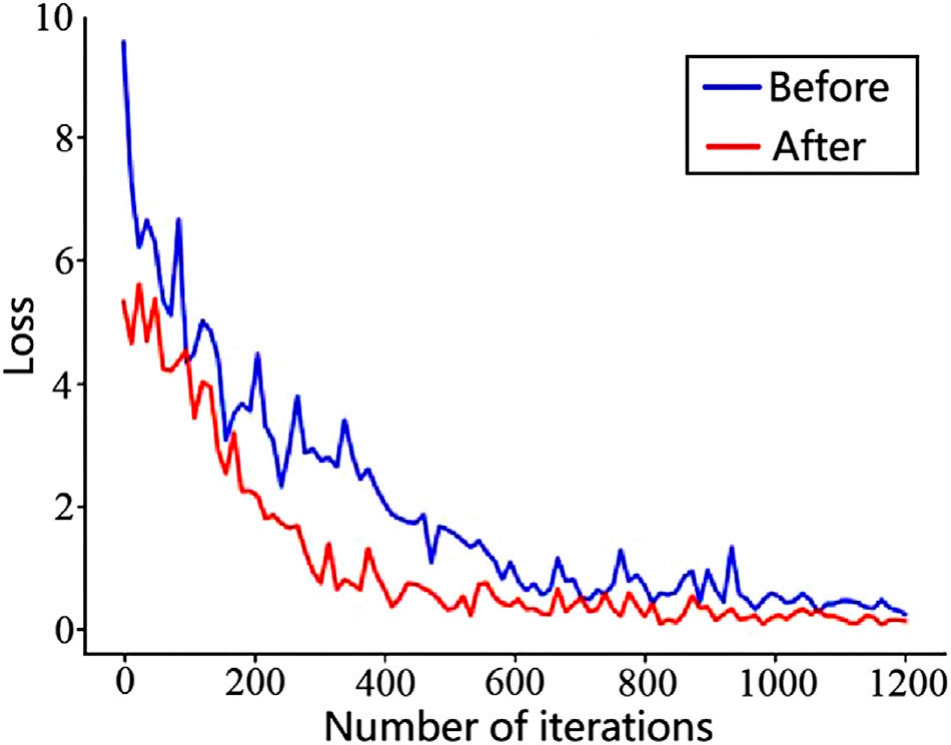
Faster R-CNN’s loss function line graph before and after optimization for logical element training.

Figure 8 shows the loss function graphs of the original faster R-CNN and the improved faster R-CNN, and the loss value was taken once every 30 iterations. The blue curve indicates the loss curve of the original faster R-CNN, and the red curve indicates the loss curve of the improved faster R-CNN. It is obvious that the loss function value in the improved faster R-CNN was lower than that of the original one, and the red curve tended to stabilize earlier than the blue curve with the number of iterations increasing. The anchor frame in the improved model was set according to the industrial control logic graph SAMA dataset, which helped the detection frame of the model find the real frame quickly during the training process, so the convergence rate is faster.

## Conclusion

In this study, the faster R-CNN target detection algorithm was improved for enhancing the efficiency of industrial control logic graph tuples recognition and achieving batch transformation of logic graph verification. The dataset was constructed and enhanced with existing industrial control logic graphs. The ResNet101 network was selected to replace the traditional VGG16 network, which enriched the image features in the deep network. K-means clustering analysis was used to set the appropriate anchor frame scale and proportion according to the scale ratio of the graph elements in the dataset. The NMS algorithm was improved to avoid accidentally deleting the candidate boxes due to the proximity of the elements. The shape similar to logic graph elements was distinguished *via* the inherent text properties in the associated elements. Comparing the improved faster R-CNN with other detection algorithms such as YOLO_V3 and the original faster R-CNN, the improved faster R-CNN algorithm had better effects in the detection of industrial logic problem elements and the detection accuracy was improved by 6.6%, while the detection speed is also greatly improved.

## Data Availability

The datasets presented in this article are not readily available because it is only for this article. Requests to access the datasets should be directed to Shilin Wu, 1995011@wtu.edu.cn.
